# Acute kidney injury outcomes in covid-19 patients: systematic review and meta-analysis

**DOI:** 10.1590/2175-8239-JBN-2022-0013en

**Published:** 2022-07-15

**Authors:** Beatriz Moreira Silva, Letícia Cristina Silva De Assis, Maurício De Castro Batista, Naara Affonso Philipps Gonzalez, Samuel Benni Dos Anjos, Miguel Angelo Goes

**Affiliations:** 1 Universidade de Mogi das Cruzes, Mogi Das Cruzes, SP, Brasil; 2 Universidade Federal de São Paulo, São Paulo, SP, Brasil

**Keywords:** SARS-CoV-2, COVID-19, Acute Kidney Injury, Renal Replacement Therapy, SARS-CoV-2, COVID-19, Injúria Renal Aguda, Terapia de Substituição Renal

## Abstract

**Background:**

Acute kidney injury (AKI) is a frequent complication of coronavirus-19 disease (COVID-19). Therefore, we decided to perform a systematic review and meta-analysis with data from the literature to relate the development of COVID-19 associated-AKI with comorbidities, medications, and the impact of mechanical ventilation.

**Methods:**

We performed a systematic review using the Newcastle-Ottawa scale and a meta-analysis using the R program. Relevant studies were searched in the PubMed, Medline, and SciELO electronic databases. Search filters were used to include reports after 2020 and cohort studies.

**Results:**

In total, 1166 articles were identified and 55 English-written articles were included based on the risk of bias. Of all COVID-19-hospitalized patients presenting with AKI (n = 18029) classified as Kidney Disease Improving Global Outcomes stage 1 to 3, approximately 18% required mechanical ventilation and 39.2 % died. Around 11.3% of the patients required kidney replacement therapy (KRT) and of these, 1093 died and 321 required continuous KRT. Death is more frequent in individuals with AKI [OR 6.03, 95%CI: 5.73-6.74; p<0.01]. Finally, mechanical ventilation is an aggravating factor in the clinical conditions studied [OR 11.01, 95%CI: 10.29-11.77; p<0.01].

**Conclusion:**

Current literature indicates AKI as an important complication in COVID-19. In this context, we observed that comorbidities, such as chronic kidney disease and heart failure, were more related to the development of AKI. In addition, mechanical ventilation was seen as an aggravating factor in this scenario.

## Resumo

**Antecedentes:**

Lesão renal aguda (LRA) é uma complicação frequente da doença do coronavírus-19 (COVID-19). Desta forma, decidimos realizar uma revisão sistemática e uma metanálise com dados da literatura para relacionar o desenvolvimento de LRA associada à COVID-19 com comorbidades, medicamentos e o impacto da ventilação mecânica.

**Métodos:**

Realizamos uma revisão sistemática usando a escala de Newcastle-Ottawa e uma metanálise utilizando o programa R. Estudos relevantes foram pesquisados nos bancos de dados eletrônicos PubMed, Medline e SciELO. Foram utilizados filtros de pesquisa para incluir relatos após 2020 e estudos de coorte.

**Resultados:**

No total, foram identificados 1166 artigos, e foram incluídos 55 artigos escritos em língua inglesa com base no risco de viés. De todos os pacientes hospitalizados por COVID-19 apresentando LRA (n = 18029) classificados como *Kidney Disease Improving Global Outcomes* estágios 1 a 3, aproximadamente 18% necessitaram de ventilação mecânica e 39,2% foram a óbito. Cerca de 11,3% dos pacientes necessitaram de terapia renal substitutiva (TRS) e destes, 1093 foram a óbito e 321 necessitaram de TRS contínua. O óbito é mais frequente em indivíduos com LRA [OR 6,03; IC95%: 5,73-6,74; p<0,01]. Por fim, a ventilação mecânica é um fator agravante nas condições clínicas estudadas [OR 11,01; IC95%: 10,29-11,77; p<0,01].

**Conclusão:**

A literatura atual indica a LRA como uma complicação importante na COVID-19. Neste contexto, observamos que comorbidades, como doença renal crônica e insuficiência cardíaca, estiveram mais relacionadas ao desenvolvimento de LRA. Além disso, a ventilação mecânica foi vista como um fator agravante neste cenário.

## Background

The coronavirus (CoV) is part of a pathogenic family of enveloped RNA viruses that can cause severe respiratory infections associated with a high mortality rate^1,2^. Recently, some CoVs have caused epidemics and pandemics, such as severe acute respiratory syndrome (SARS), Middle East respiratory syndrome, and, most recently, severe acute respiratory syndrome coronavirus-2 (SARS-CoV-2), which causes coronavirus-19 disease (COVID-19).

COVID-19 can trigger inflammatory processes, such as the release and increase of inflammatory cytokines, which can infiltrate the upper respiratory tract and lungs and cause injuries, lung parenchyma destruction, and severe inflammation^1^.

Furthermore, there is increasing evidence of kidney dysfunction in COVID-19 patients^3-5^. Although other mechanisms are being discovered^6^, the current thought is that the effects on various organs are potentially attributed to the wide distribution of the angiotensin-converting enzyme receptor 2 (ACE-2), which allows the SARS-CoV-2 virus to adhere to the cell^3,6-8^. To date, ACE-2 expression has been identified in lung, liver, stomach, ileum, colon, esophagus, and kidney cells^8^.

The kidney is one of the organs most affected by SARS-CoV-2 in severely ill patients. Acute kidney injury (AKI) from SARS-CoV-2 infection is common and sometimes results in the need for kidney replacement therapy (KRT), as required in other causes of AKI ^9,10^.

AKI occurs in 5 to 15 % of hospitalized patients with COVID-19, and mortality increases proportionally to the severity of kidney injury, especially in stages II and III of KDIGO criteria^11-12^. Recent studies have shown that most inpatients with COVID-19 who progressed to AKI requiring RRT have higher mortality. This suggests that impaired kidney function contributes to the worsening of clinical conditions and mortality in COVID-19 patients^11-13^.

The mechanism of action of the virus in the renal system remains uncertain. It is debated whether SARS-CoV-2 interacts with the renin-angiotensin-aldosterone system, enters the host cell to utilize the genetic machinery, and ultimately results in viral replication, inflammation, and cell damage^13,14^.

Despite several studies, the exact pathophysiological mechanism of COVID-19-induced AKI has not been fully elucidated. AKI as a result of ischemic acute tubular necrosis is thought to be related to respiratory failure in the crosstalk functionality, which is usually associated with systemic collapse.^15^ Moreover, researchers have reported that proteinuria and hematuria are associated with a high mortality rate in COVID-19 patients^16^.

AKI is common among critically ill COVID-19 patients^17-20^, of which 20 to 40% are admitted to intensive care units (ICUs)^21,25^. Possible causes of COVID-19-induced AKI include volume depletion, inflammation, hemodynamic changes, tubular damage associated with viral infection, thrombotic vascular processes, glomerular diseases, and rhabdomyolysis^26-28^. Furthermore, AKI patients with COVID-19 were more likely to require KRT than AKI patients without COVID-19^19,26^.

Kidney involvement, including urinary abnormalities and changes in kidney function, is observed in approximately 75% of COVID-19 patients. AKI acts as a risk factor for hospital mortality in these patients^2,28-32^. Therefore, this literature review examined the evolution of COVID-19 patients and the association between the disease and AKI. Specifically, this study aimed to identify the number of SARS-CoV-2-infected patients that developed AKI. Furthermore, we aimed to clarify the number of patients with COVID-19-related AKI requiring KRT and the number of deaths. Finally, we aimed to identify associations of pre-existing comorbidities, medications, and mechanical ventilation in patients with COVID-19-related AKI.

## Methods

The systematic review method was used to analyze studies suggesting a relationship between the development of AKI in the context of COVID-19. Because the outcomes of the articles included in the review are similar, a meta-analysis was performed, providing greater reliability to the results obtained from the collected data.

We conducted a sensitive search in Pubmed, MEDLINE, and Scielo platforms using the MeSH terms (COVID-19, Acute Kidney Injury, Renal Replacement Therapy) and their synonyms combined with the queries filter for observational, cohort, case series, and cross-sectional studies (Appendix 1). Articles with the MeSH keywords present in the title and with the appropriate study type (cohort) were included. Articles published before 2020, meta-analyses, and review articles were excluded. Based on these criteria, 60 articles, all written in English, were identified.

The risk of bias of the analyzed articles was determined using the Newcastle-Ottawa tool, whose main function is to assess the quality of non-randomized studies (cohort). The method used consists of the analysis of selection, comparability, and outcome. After classifying all articles according to the risk of bias, those with undefined risk (4 articles) or poor quality (1 article) were removed from the study, totaling 55 articles that were effectively analyzed.

The meta-analysis was based on the calculation of relative risk (RR), odds ratio (OR), and 95% confidence interval using the R program to determine if there was an influence of AKI on deaths and, if necessary, compare the deaths of individuals with and without kidney injury. In addition, we sought to assess through statistics whether mechanical ventilation had an effect on individuals who developed AKI during hospitalization. Only 36 articles brought conclusive statistics related to AKI and number of deaths and 20 articles assessed the association between mechanical ventilation and AKI.

In accordance with CNS resolution 510/2016^33^, research conducted exclusively with scientific texts for scientific literature review is neither registered nor evaluated by the ethics committee system (CEP/CONEP). Thus, the present study did not require local approval and processing.

## Results

**Figure 1:**
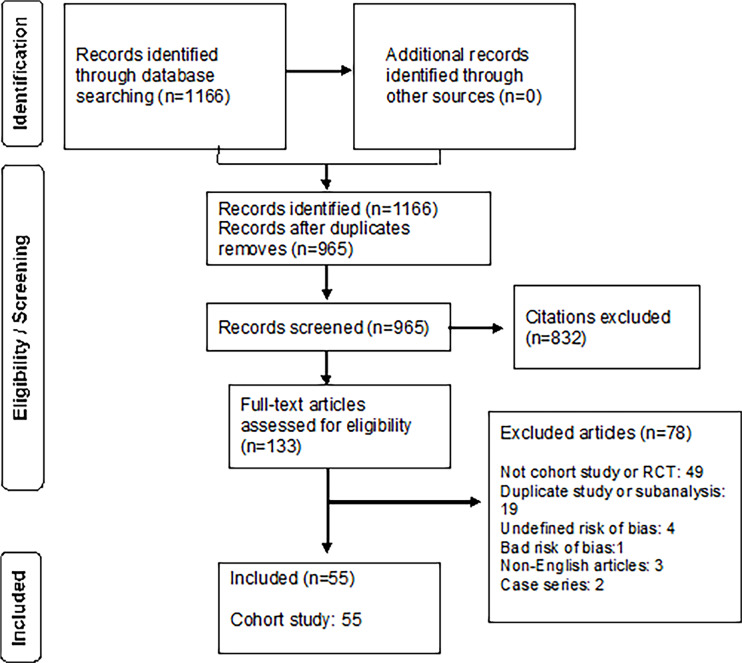
Summary of evidence, search, and selection.

In total, 1166 articles were selected by title and abstract, but only 133 met the inclusion criteria and were reviewed. After reviewing, 78 articles were excluded (Figure 1).

The results will be presented based on the data collected from the articles evaluated by risk of bias and then, by the statistical results.

Regarding the results from the Newcastle-Ottawa Quality Assessment Scale, most articles (39) achieved the maximum score, that is, one star in each of the following items: representativeness of the exposed cohort, selection of the unexposed cohort, verification of exposure, and demonstration that the outcome of interest was not present at baseline. Part of the articles (11) received 3 stars in this regard, and 5 articles did not score in the representativeness of the exposed cohort, another 5 did not score in the selection of the unexposed cohort, and 1 article did not score in the demonstration that the outcome of interest was not present at baseline. None of the selected articles scored less than 3 stars (Table 1 and 2).

**Table 1:** General information^10,14,15,17,24,25,29,31,32,34-38,40,42-48,50-79^

General information	Absolute number	Relative number
Hospitalized patients	58256	-
Acute kidney injury (AKI)	18029	30.94%
Stage 1	8067	49%
Stage 2	3478	21%
Stage 3	4925	30%
Unclassified stage	1559	8%
No acute kidney injury	40227	69.06%
AKI mortality	7068	39.2%
Non-AKI mortality	4372	10.86%
Kidney replacement therapy (KRT)	2045	11.34%
KRT mortality	1093	53.44%
Continuous kidney replacement therapy (CKRT)	321	15.6%
Mechanical ventilation (MV)	10743	18.44%
AKI	7293	67.88%
Non-AKI	3450	32.12%

**Table 2:** General information related to the risk of bias

Authors	Selection				Comparability	Outcome		
	Representativeness of the exposed cohort	Selection of the non-exposed cohort	Ascertainment of exposure	Demonstration that outcome of interest was not present at start of study	Comparability of cohorts on the basis of the design or analysis controlled for confounders	Assessment of outcome	Was follow-up long enough for outcomes to occur	Adequacy of follow-up of cohorts
Khusid et al (2020)^32^	*	*	*	*	*	*	*	*
Hirsch et al (2020)^15^	*	*	*	*	**	*	*	*
Fominskiy et al (2020)^34^	*	*	*	*	**	*	*	*
Dai et al (2021)^35^	*	*	*	*	**	*	*	*
Zheng et al (2020)^36^	*	*	*	*	*	*	*	*
Na et al (2020)^14^	*	*	*	*	**	*	*	*
Trabulus et al (2020)^37^	*	*	*	*	*	*	*	*
Kolhe et al (2020)^38^	*	*	*	*	**	*	*	*
Paek et al (2020)^39^	*	*	*	*	--	*	--	--
Wang et al (2020)^40^	--	*	*	*	**	*	*	*
Braun et al (2020)^17^	*	--	--	*	--	--	*	*
Authors	Selection				Comparability	Outcome		
	Representativeness of the exposed cohort	Selection of the non-exposed cohort	Ascertainment of exposure	Demonstration that outcome of interest was not present at start of study	Comparability of cohorts on the basis of the design or analysis controlled for confounders	Assessment of outcome	Was follow-up long enough for outcomes to occur	Adequacy of follow-up of cohorts
Stewart et al (2020)^41^	--	*	*	*	--	*	*	*
Cui et al (2020)^42^	*	--	*	*	*	*	*	*
Li et al (2020)^43^	--	*	*	*	**	*	*	*
Hussan-Syed et al (2020)^44^	*	*	*	*	**	*	*	*
Chan et al (2021)^45^	*	--	*	*	**	*	*	*
Nalesso et al (2020)^46^	*	*	*	*	**	*	*	*
Yildirim et al (2021)^47^	*	*	*	*	**	*	*	*
Ng et al (2021)^48^	*	--	*	*	**	*	*	*
Sang et al (2020)^10^	*	*	*	--	**	*	*	*
Arnold et al (2020)^49^	*	*	*	*	--	*	*	*
Russo et al (2020)^31^	*	*	*	*	*	*	*	*
Zahid et al (2020)^50^	*	*	*	*	*	*	*	*
Hamilton et al (2020)^51^	*	*	*	*	*	*	*	*
Cheng et al (2020a)^24^	*	*	*	*	*	*	*	*
Cheng et al (2020b)^29^	*	*	*	*	*	*	*	*
Costa et al (2021)^52^	*	*	*	*	**	*	--	*
Gupta et al (2021)^53^	*	*	*	*	**	*	*	*
Xu et al (2020)^54^	*	*	*	*	**	*	*	*
Khalili et al (2021)^55^	*	*	*	*	**	*	*	*
Fisher et al (2020)^25^	*	*	*	*	**	*	*	*
Charytan et al (2021)^56^	*	--	*	*	**	*	*	*
Lowe et al (2021)^57^	*	*	*	*	**	*	*	*
Piñero et al (2021)^58^	*	*	*	*	*	*	*	*
Murt et al (2021)^59^	*	*	*	*	*	*	*	*
Bowe et al (2021)^60^	--	*	*	*	*	*	*	*
Hansrivijit et al (2021)^61^	*	*	*	*	**	*	*	*
Zamoner et al (2021)^62^	*	*	*	*	*	*	*	*
Yan et al (2020)^63^	*	*	*	*	*	*	*	*
Diebold et al (2021)^64^	*	*	*	*	*	*	*	*
Tarragón et al (2021)^65^	*	*	*	*	**	*	*	*
Moledina et al (2021)^66^	*	*	*	*	--	*	*	*
Xia et al (2020)^67^	*	*	*	*	--	*	*	*
Casas-Aparicio et al (2021)^68^	*	*	*	*	**	*	*	*
Luther et al (2021)^69^	*	*	*	*	--	*	*	*
Xu et al (2021)^70^	--	*	*	*	--	*	*	*
Xiao et al (2021)^71^	*	*	*	*	--	*	--	*
Martínez-Rueda et al (2021)^72^	*	*	*	*	**	*	*	*
Basalely et al (2021)^73^	--	*	*	*	--	*	*	*
Almeida et al (2021)^74^	*	*	*	*	--	*	*	*
Doher et al (2021)^75^	*	*	*	*	**	*	*	*
Kanbay et al (2021)^76^	*	*	*	*	--	*	*	*
Strohbehn et al (2021)^77^	*	--	*	*	**	*	*	*
Alfano et al (2021)^78^	*	*	*	*	--	*	--	*
Sullivan et al (2021)^79^	*	--	*	--	--	*	--	*

Legend:

* Star; -- Undefined

In the item about comparability, the articles were rated based on the amount of information available for data analysis and results. In this regard, 26 articles received a total of 2 stars because they included patient data on age, gender, AKI stage, use of mechanical ventilation, comorbidities and KRT. Twenty-four articles scored only one star for presenting age, sex, and AKI stage, and at least one of the 3 subsequent items (Table 1 and 2).

Lastly, most articles (47) had maximum scores for outcomes. Only 3 articles were rated 2 stars, and in all these articles follow-up was not long enough for results to occur (Table 1 and 2).

**Figure 2:**
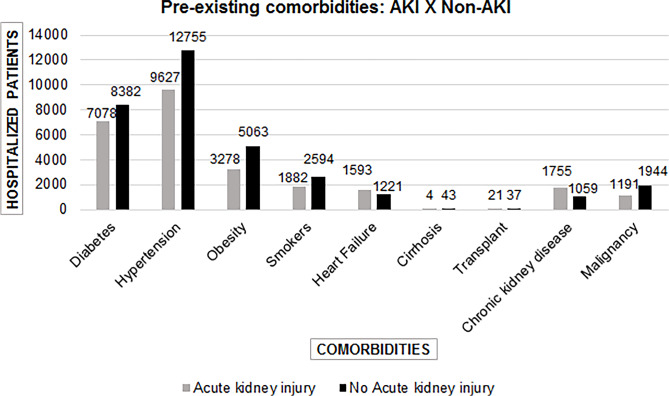
Comorbidities in patients with COVID-19 associated with acute kidney injury^10,14,15,17,24,25,29,31,32,34-38,40,42-48,50-79^.

Table 3 presents the total number of patients hospitalized for COVID-19; approximately 31% had AKI at some point during the hospital stay. COVID-19 patients with AKI were subdivided into stages based on their kidney condition (Figure 2). Stage 1 was the most common, followed by stage 3. Stage 2 had the least number of patients with no proportional distribution of AKI severity according to KDIGO (Kidney Diseases Improve Global Outcomes) criteria.

**Table 3:** Risk of bias classification

Author	Selection	Comparability	Outcome	Classification
Khusid et al (2020)^32^	^****^	^*^	^***^	Good quality
Hirsch et al (2020)^15^	^****^	**	^***^	Good quality
Fominskiy et al (2020)^34^	^****^	**	^***^	Good quality
Dai et al (2021)^35^	^****^	**	^***^	Good quality
Zheng et al (2020)^36^	^****^	^*^	^***^	Good quality
Na et al (2020)^14^	^****^	**	^***^	Good quality
Trabulus et al (2020)^37^	^****^	^*^	^***^	Good quality
Kolhe et al (2020)^38^	^****^	**	^***^	Good quality
Paek et al (2020)^39^	^****^	-	^*^	Undefined
Wang et al (2020)^40^	^***^	**	^***^	Good quality
Braun et al (2020)^17^	**	-	**	Undefined
Stewart et al (2020)^41^	^***^	-	^***^	Undefined
Cui et al (2020)^42^	^***^	^*^	^***^	Good quality
Li et al (2020)^43^	^***^	**	^***^	Good quality
Hussan-Syed et al (2020)^44^	^****^	**	^***^	Good quality
Chan et al (2021)^45^	^***^	**	^***^	Good quality
Nalesso et al (2020)^46^	^****^	**	^***^	Good quality
Yildirim et al (2021)^47^	^****^	**	^***^	Good quality
Ng et al (2021)^48^	^***^	**	^***^	Good quality
Sang et al (2020)^10^	^***^	**	^***^	Good quality
Arnold et al (2020)^49^	^****^	-	^***^	Undefined
Russo et al (2020)^31^	^****^	^*^	^***^	Good quality
Zahid et al (2020)^50^	^****^	^*^	^***^	Good quality
Hamilton et al (2020)^51^	^****^	^*^	^***^	Good quality
Cheng et al (2020a)^24^	^****^	^*^	^***^	Good quality
Cheng et al (2020b)^29^	^****^	^*^	^***^	Good quality
Costa et al (2021)^52^	^****^	**	**	Good quality
Gupta et al (2021)^53^	^****^	**	^***^	Good quality
Xu et al (2020)^54^	^****^	**	^***^	Good quality
Khalili et al (2021)^55^	^****^	**	^***^	Good quality
Fisher et al (2020)^25^	^****^	**	^***^	Good quality
Charytan et al (2021)^56^	^***^	**	^***^	Good quality
Lowe et al ( 2021)^57^	^****^	**	^***^	Good quality
Piñero et al (2021)^58^	^****^	^*^	^***^	Good quality
Murt et al (2021)^59^	^****^	^*^	^***^	Good quality
Bowe et al (2021)^60^	^***^	^*^	^***^	Good quality
Hansrivijit et al (2021)^61^	^****^	**	^***^	Good quality
Zamoner et al (2021)^62^	^****^	^*^	^***^	Good quality
Yan et al (2020)^63^	^****^	^*^	^***^	Good quality
Diebold et al (2021)^64^	^****^	^*^	^***^	Good quality
Tarragón et al (2021)^65^	^****^	**	^***^	Good quality
Moledina et al (2021)^66^	^****^	^*^	^***^	Good quality
Xia et al (2020)^67^	^****^	^*^	^***^	Good quality
Casas-Aparicio et al (2021)^68^	^****^	**	^***^	Good quality
Luther et al (2021)^69^	^****^	^*^	^***^	Good quality
Xu et al (2021)^70^	^***^	^*^	^***^	Good quality
Xiao et al (2021)^71^	^****^	^*^	**	Good quality
Martínez-rueda et al (2021)^72^	^****^	**	^***^	Good quality
Basalely et al (2021)^73^	^***^	^*^	^***^	Good quality
Almeida et al (2021)^74^	^****^	^*^	^***^	Good quality
Doher et al (2021)^75^	^****^	**	^***^	Good quality
Kanbay et al (2021)^76^	^****^	^*^	^***^	Good quality
Strohbehn et al (2021)^77^	^***^	**	^***^	Good quality
Alfano et al (2021)^78^	^****^	^*^	**	Good quality
Sullivan et al (2021)^79^	**	-	**	Bad quality

Legend:

* Star; -- Undefined

We also found that more individuals with AKI required mechanical ventilation (approximately 7300 patients, 67.88%) than those without AKI (3450 patients, 32.12%). In addition, we compared the number of deaths between patients with and without AKI. More individuals with AKI died (approximately 39,2%) than those without.

Table 3 presents the assessment of KRT in patients with AKI. KRT was administered to 2045 patients of which 53.44% died and 15.6% required CKRT. Although this information is relevant to our study, most articles did not present data regarding KRT. Therefore, it was not possible to establish a relationship regarding the efficacy of KRT in these patients.

Heart failure and chronic kidney disease were more frequent in patients with AKI than in those without (Figure 2) AKI. However, the incidence of diabetes and hypertension was higher in patients without AKI than in those with AKI. Nevertheless, these comorbidities remain significant in patients with AKI. Regarding transplanted and cirrhotic patients, nearly all articles did not present enough data to develop a hypothesis. Smoking contributed significantly to AKI development, as did obesity (Figure 2).

**Figure 3:**
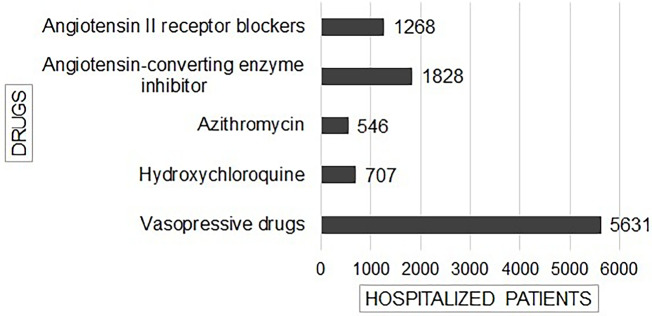
Drugs used in patients with COVID-19^10,14,15,17,24,25,29,31,32,34-38,40,42-48,50,51-79^.

Few articles reported the medications used to treat COVID-19 (Figure 3). Vasopressor drugs were most common among AKI patients (around 5631 administrations). The most common medications used chronically before SARS-CoV-2 infection were angiotensin II blockers (1268 patients) and angiotensin-converting enzyme inhibitors (1828 patients) (Figure 3). Other drugs related to AKI in COVID-19 patients were hydroxychloroquine (707 patients) and azithromycin (546 patients).

**Figure 4:**
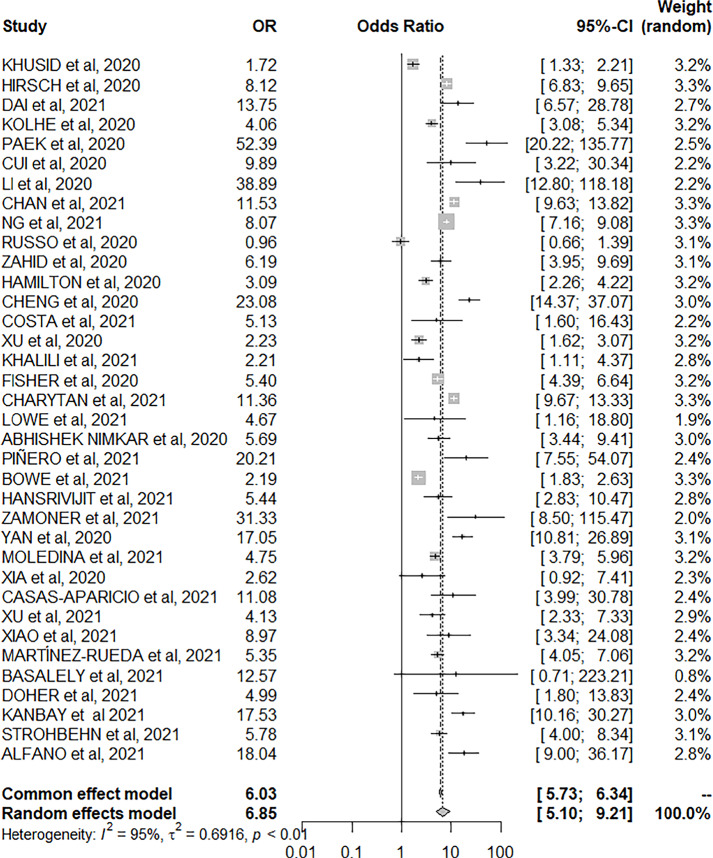
Forest plot of the association of acute kidney injury and number of deaths.

Information about the influence of AKI and the use of mechanical ventilation in patients hospitalized for COVID-19 presented in the articles was extracted and statistically analyzed. In most articles, there was a greater number of deaths in patients who presented AKI during hospitalization compared to those without AKI. In this regard, it was concluded that the chance of patients with AKI dying is greater than in individuals without AKI [OR 6.03, 95%CI: 5.73-6.74; p<0.01]. (Figure 4)

**Figure 5:**
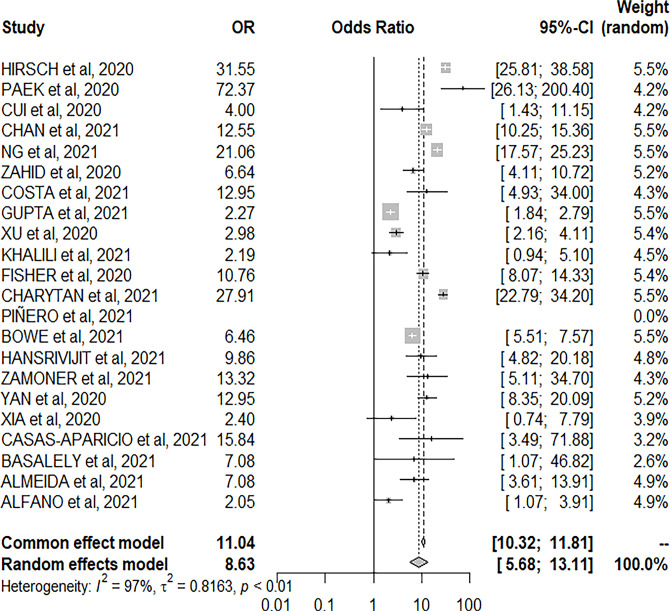
Forest plot of the association of mechanical ventilation and acute kidney injury.

Based on the data, it also appeared that mechanical ventilation is an aggravating factor for AKI, which may be related to the worsening of the clinical conditions studied [OR 11.01, 95%CI: 10.29-11.77; p<0.01] (Figure 5).

The data presented by these articles regarding drugs used during hospitalization and the possible correlation with comorbidities brought inconclusive calculations and, therefore, are not shown in the present study.

## Discussion

Regarding the cohort selection criteria, 39 articles had the highest score, meaning they included a very comprehensive cohort and the unexposed population was selected from the same community as the exposed, providing more reliable research results. Furthermore, all the articles were based on medical records and demonstrated that the outcome of interest was not present at the beginning of the analysis, ensuring reliability to the results and security of data sources. Some articles restricted the cohort to a specific group, while others did not specify whether the unexposed group was drawn from the same population as the exposed group, and only one did not make it clear whether the outcome of interest was present in the patients prior to the start of the study. However, these factors alone did not significantly affect the quality of these studies.

Regarding data comparability, fundamental criteria for evaluating the articles were established, with data specified throughout the texts of the selected studies. These data included: age, sex, AKI stage (one star), mechanical ventilation, presence of comorbidities, and the need for KRT (one star). Most articles showed all the data that are extremely useful for a holistic analysis of the clinical picture of patients hospitalized for COVID-19 and also allow for greater correlations between the data. A significant part of the articles failed with some of the items in the second group of data, and most of these did not show data regarding the need for KRT in individuals with AKI. This made it impossible for deeper analysis of the clinical impact of COVID-19 on AKI, but it did not compromise the other correlations that were necessary to carry out this study.

As for the last criterion, which deals with the outcomes, most of the articles had a maximum score of 3 stars, as the results of these studies were compared based on patient records (one star), the patient follow-up was appropriate (one star), and all or almost all aspects were considered in the follow-up (one star). This made it possible to compare the data between the articles and, consequently, to discuss the possible cause-and-effect relationships found in the clinical and pathological conditions of the patients. All of articles that did not reach the total score did not follow-up long enough to observe the results after the treatment of the diseases, making it impossible to analyze a wider spectrum of the effects of AKI on COVID-19patients. However, these articles were maintained due to the richness of data and their inclusion did not compromise the analysis of the other articles. Patient data was compiled from the included articles and then the association with possible clinical repercussions arising from AKI development was assessed. We sought to establish criteria to analyze comorbidities, kidney diagnoses correlated with COVID-19, and clinical outcomes (continuous treatment with KRT or death) of the patients. However, most articles did not provide essential data concerning the clinical course of patients with a good prognosis after renal replacement therapy treatment.

All data listed in this study reinforced that AKI is one of the complications that most frequently affect patients admitted to the ICU. Among patients hospitalized for COVID-19, AKI showed an important prevalence, being an aggravating factor for the clinical condition of these patients and considerably increasing the mortality rate.

Mechanical ventilation in COVID-19 patients is one of the main therapeutic resources used^80^. Mechanical ventilation can cause high intrathoracic pressure with decreased cardiac output and decreased kidney perfusion, with consequent injury to renal tubular cells^81^. In this context, therefore, statistical analysis allowed to establish the relationship between mechanical ventilation and COVID-19-related AKI. In fact, the present study showed that mechanical ventilation is a risk factor for the development of AKI in patients infected with SARS-CoV-2.

Regarding comorbidities, the present study showed a relationship between the presence of chronic diseases and the risk of AKI in patients with COVID-19. An individual with diabetes mellitus and arterial hypertension for six months increases the risk of developing AKI. The drugs used must also be evaluated for potential nephrotoxicity, and, when necessary, doses should be adjusted to avoid kidney damage^82^.

In addition, smoking and obesity were among the predisposing factors for a worse prognosis in these patients. Studies have shown that tobacco smoke contains more than 4000 particles and gases, some of which are nephrotoxic^83^. Obesity also has an influence^84^. Although this relationship can be found within the studies, the lack of data in most articles made it impossible to carry out a precise statistical calculation, which demonstrates the need for more effective and detailed data collection in future studies.

Concerning to the drug therapy used in patients with COVID-19, the study showed associations between the mechanisms of certain drugs and pathogenesis of the virus, regarding the worsening of clinical condition.

Previous studies demonstrated that approximately 70% of patients who started treatment with vasopressor drugs developed AKI during therapy^85^. Therefore, as SARS-CoV-2 uses ACE-2 to penetrate the cell, one study suggested that treatment with ACE inhibitors or angiotensin-2 receptor blockers may increase the risk of serious complications associated with COVID-19^86^, but this is not supported by other researchers. However, like the presentation of comorbidities, the presentation of drugs used during the patients’ hospitalization was not effective, making statistical calculation difficult and requiring further studies on this topic.

## Conclusion

Our study found that SARS-CoV-2 infection was related to AKI development and that AKI is a relevant complication in COVID-19 hospitalization cases. However, it is also worth emphasizing that comorbidities may be related to more severe cases of AKI. Furthermore, the number of deaths was considerably higher among individuals with AKI than those without. It is also important to mention that mechanical ventilation can be an aggravating factor and should be treated with caution.

The included articles did not provide sufficient data to conclude on the efficacy of KRT and drug treatments. Besides, most articles did not provide essential data concerning the clinical course of patients with a good prognosis after KRT and nearly all articles did not present enough data to establish a hypothesis about transplanted and cirrhotic patients with kidney commitment. Consequently, randomized and controlled clinical trials and prevalence and incidence studies are necessary to analyze all potential influencing factors correlated with AKI progression in SARS-CoV-2-infected patients.

## Supplementary Material

The following online material is available for this article:

Appendix 1 - Search Strategy.
