# Supplementary material for: Acute kidney injury outcomes in covid-19 patients: systematic review and meta-analysis
Source: J Bras Nefrol. 2022 Jul 15;44(4):543–56. doi: 10.1590/2175-8239-JBN-2022-0013en (PMC9838673; doi:10.1590/2175-8239-JBN-2022-0013en)
Supplement: Supplementary file 1 [file 0101-2800-jbn-2022-0013-suppl01.pdf]

Braz. J. Nephrol. (J. Bras. Nefrol.)
https://doi.org/10.1590/2175-8239-JBN-2022-0013en

Supplementary Material to "Acute kidney injury outcomes in covid-19 patients: systematic review and meta-analysis"

Appendix 1 - Search Strategy.

Database	Search Strategy
	#1 "SARS-CoV-2" OR "COVID-19" [MeSh] OR "Acute Kidney Injury" [MeSh] OR "Renal Replacement Therapy" [MeSh] OR "Acute Kidney Disease" [MeSh] OR "Acute Renal Failure" [MeSh]
Medline	#2 Cohort study
Pubmed	#3 Cohort study
Scielo	#4 Cohort study
